# “Pulsating proptosis and heavy eye syndrome precipitated by neurofibromatosis type 1: A case report”

**DOI:** 10.1097/MD.0000000000027575

**Published:** 2021-10-22

**Authors:** Yi-Fen Lai, Lung-Chi Lee, Yi-Hao Chen, Ke-Hung Chien

**Affiliations:** Department of Ophthalmology, Tri-Service General Hospital, National Defense Medical Center, Taipei, Taiwan.

**Keywords:** neurofibromatosis type 1, orbital decompression, pulsating exophthalmos, strabismus

## Abstract

**Rationale:**

Neurofibromatosis type 1 (NF1) is a hereditary disease characterized by café-au-lait spots, peripheral neurofibromas, Lisch nodules, optic nerve glioma, and sphenoid wing dysplasia. Pulsating proptosis is associated with a sphenoid bony defect. Heavy eye syndrome is characterized by acquired esohypotropia in patients with high myopia. This study aimed to describe the presentation of pulsating proptosis and heavy eye syndrome precipitated by NF1 and its management.

**Patient concerns:**

A 41-year-old woman presented with progressive pulsating proptosis and hypodeviation of the right eye over the past 2 years. The axial length of the right eye was 36.81 mm. The right eye presented with esohypotropia and hypoglobus. The ocular motility examination showed limitations in all directions, especially in supraduction. Brain computed tomography revealed sphenoid wing dysplasia of the right orbit. The meningocele protruded through the orbital defect, lifting the globe. Brain magnetic resonance imaging demonstrated superior rectus muscle (SR) medial displacement and lateral rectus muscle inferior displacement. Physical examination revealed café-au-lait macules and neurofibromas on the trunk.

**Diagnosis:**

NF1 with pulsating proptosis and heavy eye syndrome.

**Interventions:**

The patient declined neurosurgery due to risk and economic reasons. To manage her main concern regarding cosmetics, we performed orbital floor decompression, SR resection with advancement, maximal hang-back recession of the inferior rectus muscle, and a partial Jensen's procedure.

**Outcomes:**

Proptosis was reduced. The eye position became more symmetrical. The range of eye movements was also increased.

**Lessons:**

This case describes a rare synchronous presentation of pulsating proptosis and heavy eye syndrome precipitated by NF1. Adult-onset presentation implied a progressive process in NF1. The case also showed a different etiology from that of typical heavy eye syndrome. It reminds ophthalmologists that orbital imaging should be performed in high myopia patients with strabismus to evaluate the extraocular muscle pathway. Furthermore, the case demonstrated a management that avoided the risk and expensive cost of neurosurgery, which has not been reported.

## 1 Introduction

Neurofibromatosis type 1 (NF1) is known as von Recklinghausen's disease, an autosomal dominant hereditary neurodermal dysplasia, which is characterized by café-au-lait spots, peripheral neurofibromas, Lisch nodules, optic nerve glioma, and sphenoid wing dysplasia.^[1]^ Sphenoid wing dysplasia is uncommon and occurs only in 4% to 11% of NF1 cases.^[2]^ Pulsating proptosis is associated with a sphenoid bony defect. Moreover, clinical diagnosis of NF1 is confirmed when patients meet at least 2 of the 7 criteria described by the National Institutes of Health (see Table S1, Supplemental Digital Content, which demonstrates the National Institutes of Health criteria for the diagnosis of NF1).^[3]^ Heavy eye syndrome is common in patients with high myopia, resulting in acquired eso-hypotropia, with limited supraduction and abduction.^[4]^ The elongated globe herniates superotemporally between the superior rectus muscle (SR) and lateral rectus muscle (LR), resulting in SR medial displacement and LR inferior displacement. Therefore, inferior dislocation of the LR pulley increases infraduction, and medial dislocation of the SR pulley increases the force of adduction, leading to limited supraduction and abduction.^[5]^ However, few studies have reported both presentations in NF1. In this case report, we have described a rare synchronous presentation with pulsating proptosis and heavy eye syndrome precipitated by NF1 and its management.

## 2 Case report

**Figure 1:**
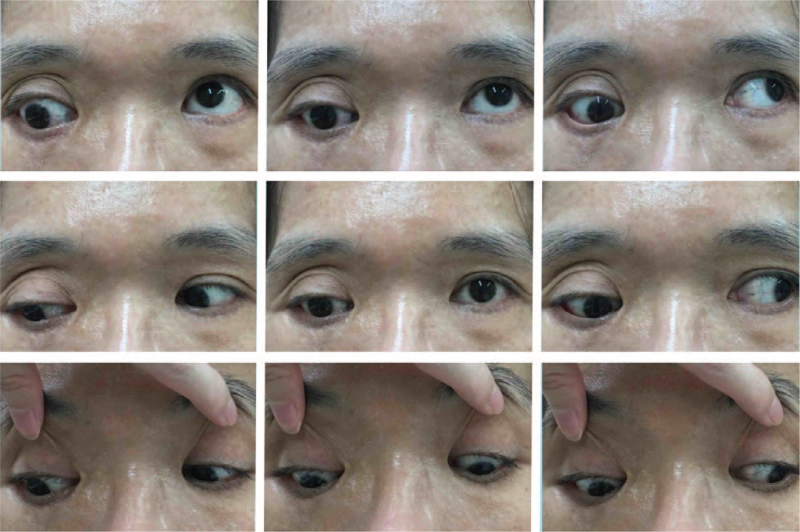
Preoperative composite nine-gaze picture showing large angle hypotropia and mild esotropia of the right eye in the primary position. Hypoglobus and proptosis of the right eye are also noted.

**Figure 2:**
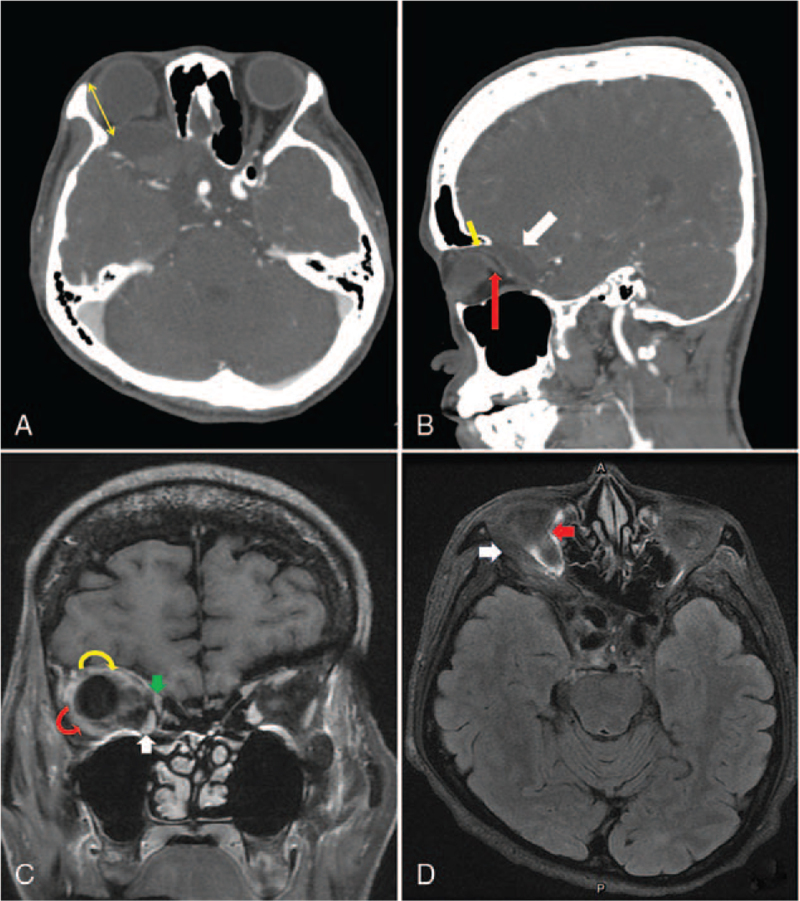
(A) A short lateral orbital wall (yellow double head arrow) and sphenoid wing dysplasia of the right orbit are noted. (B) A meningocele protruding through the orbital defect into the right orbit and lifting the globe (white arrow). The herniated meningocele compresses the posterior portion of the superior rectus muscle (SR) (yellow arrow) and optic nerve (red arrow). (C) Brain magnetic resonance imaging (MRI) showing inferior displacement of the lateral rectus muscle (LR) (red curved arrow) and medial displacement of the SR (yellow curved arrow) with a supra-temporally shifted globe. The medial rectus muscle (green arrow) and inferior rectus muscle (white arrow) are hypertrophic and crowded in the medial orbital cavity. (D) The meningocele herniates into the right orbit and compresses the LR (white arrow). The LR is shifted inferiorly and appears at the same level as the inferior rectus muscle (red arrow).

**Figure 3:**
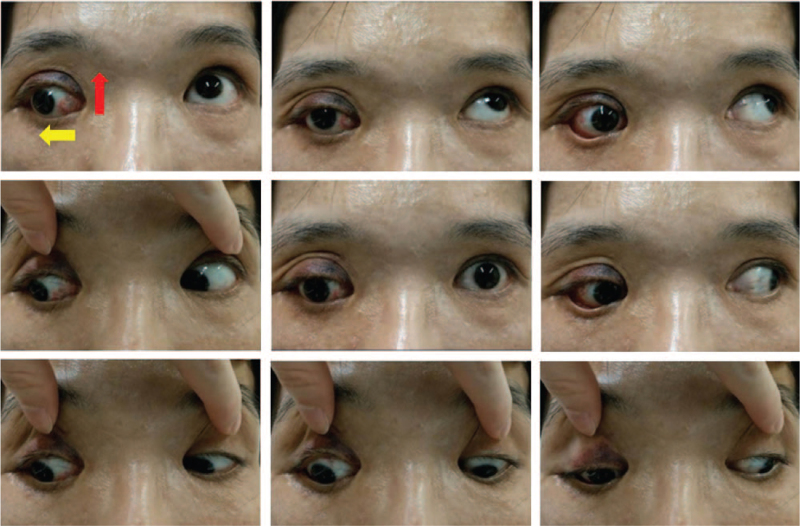
The eye position has become more symmetrical postoperatively. The range of supraduction (red arrow) and abduction (yellow arrow) has also increased.

A 41-year-old woman visited our clinic for progressive pulsating proptosis and hypodeviation of the right eye over the past 2 years. She had no pertinent medical or trauma history. The best-corrected visual acuity of the right eye was hand motion at 30 cm. Furthermore, her right eye presented with esohypotropia and hypoglobus with globe enlargement (refraction: non-measurable; axial length, 36.81 mm). The ocular motility examination of the right eye showed limitations in all directions, especially in supraduction (Fig. 1). The pulsatile rhythm of the right eye was synchronized with each heartbeat (see Supplemental Video, which demonstrates that the pulsatile rhythm of the right eye was synchronous with the sounds of electrocardiography (4 second, 925 KB)). Previous images reviewed did not reveal the presence of strabismus or proptosis. Slit lamp examination revealed Lisch nodules on the iris. Brain computed tomography (CT) revealed a short lateral orbit wall (maximum length: 2. 7 cm) and sphenoid wing dysplasia of the right orbit (Fig. 2A). A meningocele protruded through the bony defect of the sphenoid wing into the right orbit, adjacent to the globe posteriorly, and lifted it up (Fig. 2B). Brain magnetic resonance imaging revealed that the extraocular muscles were hypertrophic and crowded on the medial side of the orbital cavity. Relative inferior displacement of the LR muscle and medial displacement of the SR muscle with supra-temporal shift of the myopic globe were also noted (Fig. 2C). The posterior portion of the SR and LR were compressed by the meningocele (Figures 2B and 2D, respectively). Physical examination revealed café-au-lait skin macules (maximum diameter: 42 mm) with countless neurofibromas over the trunk. Combining the above findings with sphenoid bone dysplasia, café-au-lait skin macules, and neurofibromas, the characteristic features met the criteria of NF1 described by the National Institutes of Health. Thus, a clinical diagnosis of heavy eye syndrome and pulsating proptosis precipitated by NF1 was made. Right fronto–zygomatico–orbital craniotomy with sphenoid wing reconstruction was advised by a neurosurgeon. However, the patient declined this approach owing to the risks associated with neurosurgery and economic reasons. To manage her main concern regarding the cosmetic aspect of proptosis and strabismus, we performed orbital floor decompression accompanied by SR resection (10 mm) with advancement (3 mm anterior to the original insertion), maximal hang-back recession of the inferior rectus muscle (IR), and a partial Jensen's procedure. On the sixth postoperative week, the range of eye motion showed improvement, with residual limitation in supraduction. Proptosis was reduced (2 mm), and the eye position became more symmetrical (Fig. 3). There was no relapse or major complications, except mild irritation in the operated eye during the 8-week follow-up.

## 3 Discussion

We have described a case of a 41-year-old woman with progressive pulsating proptosis and heavy eye syndrome precipitated by NF1. The meningocele protruded through the orbital defect, lifting the globe and shifting SR and LR, thereby leading to the presentation of pulsating exophthalmos and heavy eye syndrome. In addition, we performed orbital floor decompression accompanied by SR resection with advancement, maximal hang-back recession of the IR muscle, and a partial Jensen's procedure. The procedure improved her proptosis, eye position, and range of eye movement.

In this case, ocular manifestations were interesting. Orbital defects may be congenital. However, pulsating proptosis and heavy eye syndrome occur in adulthood. This demonstrated that the disease process was progressive. Possible reasons for adult-onset presentation, such as that in our case, may be attributed to 2 causes—the progressive loss of the sphenoid wing and a progressively enlarging globe. Macfarlane et al reported an NF1 pediatric case with intact sphenoid bone that presented with sphenoid wing dysplasia 6 years later.^[6]^ Sanjay et al reported progressive thinning of the sphenoid wing and thinning transforming to gross bony defects using CT.^[2]^ Chen et al reported 3 children with neurofibroma, which became increasingly myopic with age, while the unaffected eye remained emmetropic.^[7]^ Progressive globe enlargement over time in NF1 was also noted by Morales et al.^[8]^ Based on the above studies, we hypothesized that the progressively enlarged globe and the herniated meningocele from the progressive enlarged sphenoid defect led to its clinical manifestation in adulthood.

In addition, heavy eye syndrome in our case is worthy of discussion. Our patient had esohypotropia with high axial myopia, as observed in heavy eye syndrome. The clinical manifestation was the same as that of typical heavy eye syndrome, but the underlying etiology was different. The cause of typical heavy eye syndrome is degeneration of the SR–LR band. However, the main cause of the limited extraocular movement in our case was the shifted extraocular muscle pathways due to meningocele compression, as evidenced on imaging studies. The herniated meningocele displaced the posterior portion of the SR and LR. A previous study also reported a similar etiology.^[9]^ Our patient was more myopic than those previously reported. Our case suggests that the esotropia–hypotropic complex in high myopia can occur in cases other than those with the typical etiology of heavy eye syndrome. Orbital imaging should be performed in patients with high myopia with strabismus to evaluate the extraocular muscle pathway.

Reconstruction of sphenoid bone dysplasia has been successfully described by the placement of a titanium mesh or split calvarial bone in several case reports.^[10]^ As our patient refused neurosurgery and requested cosmetic improvement, we performed orbital decompression to restore the volume of the orbit. A previous study has reported that lateral wall decompression is favored in patients with high axial myopia.^[11]^ However, this was not suitable for our case. The later orbital wall in our patient was relatively short. Hence, the effect of lateral wall decompression is limited. Decompression of the lateral wall might also increase the risk of brain damage and extrusion from the decompression site. In our case, the advantages of using orbital floor decompression were preservation of space on the long axis for the globe after repositioning the soft tissue and a lesser safety concern. The disadvantage of the procedure is a higher risk of hypoglobus,^[12]^ which could be avoided with limited bony damage to the anterior orbit floor. In our case, the postoperative extent of hypoglobus did not increase. In addition, we performed traditional recession–resection surgery using SR resection with advancement and maximal hang-back recession of the IR. To return the dislocated globe into the muscle cone and normalize the anatomy of the SR and LR, we also performed loop myopexy using a partial Jensen's procedure, which is performed by lifting the SR and LR and then splitting the muscle in half from the insertion past the equator. Loop myopexy with partial Jensen's procedure was performed by suturing the lateral half of the SR and the superior half of the LR together with a 5-O non-absorbable suture at 14 mm from the limbus. During the postoperative period, the extraocular movement showed improvement in all directions, with residual limitation in supraduction. In addition, the eye position became more symmetrical at the primary position, and proptosis was also reduced.

The present study has several strengths. First, we describe a unique synchronous presentation of heavy eye syndrome and pulsatile proptosis precipitated by NF1, which has rarely been reported in the literature. Second, the case demonstrated a different etiology of heavy eye syndrome other than degeneration of the SR–LR band. Third, the management in the present study improved the patient's cosmetic appearance. Moreover, the procedure avoided the risk and cost of neurosurgery, which has not been reported. Nonetheless, this case report has several limitations. Management ameliorating the cosmetic problem cannot extinguish pulsation. Although the eye position became more symmetrical in the primary position, residual limitation in supraduction was still noted. Thus, management in future studies may result in better results.

## 4 Conclusion

We have reported a rare case of a 41-year-old woman with progressive pulsating proptosis and heavy eye syndrome precipitated by NF1. The case demonstrated the progression of NF1, different etiology from that of a typical heavy eye syndrome, and the management avoiding the risk and expensive cost of neurosurgery. It reminds ophthalmologists that orbital imaging should be performed in high myopia patients with strabismus to evaluate the extraocular muscle pathway. In addition, the management improved the patient's strabismus and cosmetic problems, which has not been reported.

## Author contributions

**Conceptualization:** Yi-Fen Lai, Lung-Chi Lee, Ke-Hung Chien.

**Data curation:** Yi-Fen Lai, Lung-Chi Lee.

**Formal analysis:** Yi-Fen Lai, Lung-Chi Lee.

**Funding acquisition:** Ke-Hung Chien.

**Investigation:** Yi-Fen Lai, Lung-Chi Lee.

**Methodology:** Lung-Chi Lee, Ke-Hung Chien.

**Project administration:** Yi-Hao Chen, Ke-Hung Chien.

**Resources:** Yi-Hao Chen, Ke-Hung Chien.

**Supervision:** Lung-Chi Lee, Yi-Hao Chen, Ke-Hung Chien.

**Validation:** Lung-Chi Lee, Yi-Hao Chen, Ke-Hung Chien.

**Writing – original draft:** Yi-Fen Lai.

**Writing – review & editing:** Lung-Chi Lee.

## Supplementary Material

Supplemental Digital Content

## Supplementary Material

Supplemental Digital Content
