# Supplementary material for: “Pulsating proptosis and heavy eye syndrome precipitated by neurofibromatosis type 1: A case report”
Source: Medicine (Baltimore). 2021 Oct 22;100(42):e27575. doi: 10.1097/MD.0000000000027575 (PMC8542118; doi:10.1097/MD.0000000000027575)
Supplement: Supplemental Digital Content [file medi-100-e27575-s001.docx]

Supplemental digital content Table that demonstrates the National Institutes of Health criteria for the clinical diagnosis of neurofibromatosis type 1.

| National Institutes of Health criteria for the clinical diagnosis of neurofibromatosis type 1 | |
| --- | --- |
| 1 | ≥ 6 café-au-lait skin macules >5mm in prepubertal individuals and >15 mm in postpubertal individuals |
| 2 | Axillary or inguinal freckling |
| 3 | ≥2 neurofibromas of any type or one plexiform neurofibroma |
| 4 | ≥ 2 Lisch nodules |
| 5 | Optic glioma |
| 6 | Bone lesion with sphenoid dysplasia or thinning of the long bone cortex with or without pseudoarthrosis |
| 7 | A first-degree relatives(parent, sibling or offspring) that meets National Institutes of Health criteria |
| The diagnosis of neurofibromatosis type 1 requires two or more of the above criteria. | |
